# Classifying Aging As a Disease: The Role of Microbes

**DOI:** 10.3389/fgene.2016.00212

**Published:** 2016-12-01

**Authors:** Michael S. Lustgarten

**Affiliations:** Jean Mayer USDA Human Nutrition Research Center on Aging, Tufts UniversityBoston, MA, USA

**Keywords:** aging, inflammation, oxidative stress, insulin resistance, telomere shortening, Alzheimer's disease, cardiovascular disease (CVD), cancer

Recent publications have proposed that aging should be classified as a disease (Bulterijs et al., 2015; Zhavoronkov and Bhullar, 2015; Zhavoronkov and Moskalev, 2016). The goal of this manuscript is not to dispute these claims, but rather to suggest that when classifying aging as a disease, it is important to include the contribution of microbes.

As recently as ~115 years ago, more than half of all deaths were caused by infectious diseases, including pneumonia, influenza, tuberculosis, gastrointestinal infections, and diphtheria (Jones et al., 2012). Since then, the establishment of public health departments that focused on improved sanitation and hygiene, and the introduction of antibiotics and vaccines allowed for a dramatic decrease in infectious disease-related mortality (Report, 1999). In 2010, the death rate for infectious diseases was reduced to 3% (Jones et al., 2012). Simultaneously, as infectious disease-related mortality rates have decreased, global lifespan has increased from ~30 to ~70 years (Riley, 2005).

Because death rates due to infectious diseases have been reduced to very low levels, we've forgotten about the adverse effects of microbes on our existence. The fact is, we live in a microbial world. Although there are currently ~7 billion people, in contrast, the total number of prokaryotes and viruses have been estimated at 10^30^ and 10^31^, respectively (Whitman et al., 1998; Duerkop et al., 2014). Even without including other microbes (e.g., fungi, protozoa), humans are outnumbered by more than 10^21^ to 1! All of these microorganisms aren't detrimental to human health, but more than 1400 microbial species have been shown to be pathogenic (Taylor et al., 2001).

Even at a young chronological age, microbes find their way into the blood and tissues. Circulating microbial DNA is found in young, healthy adults (average age, BMI: 21 years < 25 kg/m^2^) (Païsse et al., 2016). Interestingly, levels of circulating bacterial DNA were not homogeneous: some subjects had 3-fold or more circulating bacterial DNA when compared with others. Moreover, various bacterial species are found in skeletal muscle, heart, liver, adipose tissue, and in the brains of young mice (Lluch et al., 2015).

With the passage of time, the barriers responsible for keeping microbes out of us weaken. For example, tight junctions (TJs) connect epithelial cells, thereby minimizing the space in between the cells, and minimizing the ability of microbes to translocate into the blood. TJs are comprised of proteins such as junctional adhesion molecule (JAM), zonulin (e.g., ZO-1), occludins, and claudin. Bacteria and viruses, including *Vibrio cholera, Shigella*, and rotavirus have evolved mechanisms to impair TJ assembly: *V*. *cholera* uses a cleavage product from the ZOT protein to dissociate ZO-1 from the cell periphery, ZO-1 binds to the actin-containing tail of *Shigella*, thereby disrupting the TJ, and rotavirus uses the VP8 fragment of VP4 to dissociate claudin, occludin, and ZO-1 (Guttman and Finlay, 2009). Whether caused by pathogenic microbes or because of defects in host gene expression, levels of many of these tight junction proteins, including JAM-A, ZO-1, and occludin are decreased in old, when compared with young (Tran and Greenwood-Van Meerveld, 2013). Furthermore, although the immune system should protect us against an increase in microbial burden, however, many aspects of the immune response are decreased, whereas others are increased, thereby resulting in dysregulation. This phenotype is known as immunosenescence (Pera et al., 2015). Interestingly, a causative role for microbes on reducing immune function is suggested by the finding that young adults infected with cytomegalovirus (CMV) exhibit signs of immunosenescence (Turner et al., 2014).

The impact of decreased barrier function and immunosenescence would be expected to lead to an increase in circulating microbes in old, when compared with young. Although circulating levels of bacterial DNA have yet to be reported in older adults, plasma levels of lipopolysaccharide (LPS), which is found in the outer membrane of gram-negative bacteria, and levels of the receptors that bind to LPS (TLR4) and to bacterial flagellin (TLR5), are elevated in older adults, when compared with young (Qian et al., 2012; Ghosh et al., 2015). In line with this, the incidence of bloodstream infections with LPS-containing *Escherichia coli* is increased by more than 10-fold in adults older than 74, when compared with subjects younger than 50 years (Williamson et al., 2013). Similarly, the incidence of bloodstream infections with gram-positive bacteria (*Staphylococcus aureus*) is elevated by more than 8–17 fold in older adults (Klevens et al., 2007).

What are the consequences of an age-related increase in microbial burden? Microbes and/or microbial products are causatively involved in multiple theories of aging, including insulin resistance, oxidative stress, inflammation, and telomere shortening. In support of this, LPS injection into young, healthy subjects (average age, BMI: 26 years, < 25 kg/m^2^) causes insulin resistance, as determined by elevated HOMA-IR values (Mehta et al., 2010). Oxidative stress is increased in response to the binding of LPS and bacterial flagellin to their respective receptors (Yuan et al., 2013; Kim et al., 2016). Levels of the pro-inflammatory cytokines IL-6 and TNF-α are increased when LPS binds to TLR4 (Greenhill et al., 2011). Telomere shortening occurs at a faster rate in the presence of CMV infection (Parry et al., 2016). Interestingly, the prevalence of CMV infection increases from ~20% in adults younger than 50 years, to ~40% in 50–70 year olds, to 100% in adults older than 70 (Parry et al., 2016). Collectively, these data support a causative role for microbial burden on mechanisms that have been commonly hypothesized to drive the aging process.

Microbial burden is also involved in mechanisms related to age-related disease, including cardiovascular disease (CVD), Alzheimer's disease, cancer, stroke, and diabetes. In support of this, approximately 10-fold more circulating bacterial DNA is found in CVD patients, when compared with healthy controls (Dinakaran et al., 2014). Oral and gut microbes are found within atherosclerotic plaque (Koren et al., 2011). A causative role for bacteria on cardiovascular disease is supported by the finding that the oral microbe, *Porphyromonas gingivalis*, causes atherosclerotic plaque formation (Velsko et al., 2014).

Moreover, many of the molecular players related to atherosclerotic plaque formation, including C-reactive protein (CRP), LDL cholesterol, and fibrinogen (Spronk et al., 2004), are protective against infection-related mortality. For example, although injection of *Streptococcus pneumonae* kills 80% of wild type mice within 2 days, survival is improved by more than 4-fold in the presence of CRP (Mold et al., 2002). Similarly, although infection with *Klebsiella pneumoniae* kills all wild type mice within 2 days, survival is significantly improved in mice that are genetically engineered to have elevated circulating levels of LDL cholesterol (Netea et al., 1996). Interestingly, LDL acts as a carrier for LPS (Levels et al., 2005), and is involved in mechanisms that inactivate LPS (Weinstock et al., 1992). Approximately half of all fibrinogen-deficient mice that are injected with *S. aureus* die within 12 days, whereas mice that have wild type levels of fibrinogen are protected (Prasad et al., 2015). In addition, fibrinogen levels are significantly correlated with the amount of bacteria found in blood (Amar et al., 2011). CRP, LDL, and fibrinogen increase during aging (Abbott et al., 1983; Kannel et al., 1987; Herman et al., 2009), findings that support a link between microbial burden with the progression of CVD.

Patients with Alzheimer's disease (AD) have an increased amount of circulating bacteria (*spirochaetes*) when compared with age-matched, AD-free controls (Miklossy, 1993). Four-fold more *Treponema* bacteria, 3-fold more herpes simplex virus type I DNA, and fungi, including *Candida albicans* and *Sacharomyces cerevisiae* have been found in the brains of AD patients, when compared with age-matched controls (Riviere et al., 2002; Wozniak et al., 2009; Pisa et al., 2015). Further support for the hypothesis that Alzheimer's disease has a microbial component comes from the finding that β-amyloid protein (Aβ), which is a key mediator of AD pathology, is an antimicrobial peptide. Aβ inhibits the growth of various fungi and bacteria, when compared against the well-known anti-microbial peptide, LL-37 (Soscia et al., 2010). In addition, levels of another antimicrobial peptide, β-defensin-1, are elevated in AD brains, when compared with age-matched controls (Williams et al., 2013). These data suggest that the increased prevalence of Aβ and β-defensin-1 that are found in the brains of AD patients may be a compensatory mechanism to protect the brain against microbial infection.

A role for microbial burden on cancer incidence is supported by the findings that liver tumor amounts and tumor size are significantly increased in the presence of LPS (Dapito et al., 2012), and that exposure to LPS causes pancreatic cancer. Mice treated with LPS develop significantly more advanced pancreatic intraepithelial neoplasias, when compared with untreated controls (Ochi et al., 2012). Beyond LPS, bacteria, viruses, and parasites are associated with or are causatively involved with the progression of various cancers, including *H. pylori* with stomach cancer, the hepatitis B and C viruses with liver cancer, the human papillomavirus virus (HPV) with cervical cancer, and the parasite *Schistosoma haematobium* with bladder cancer (de Martel et al., 2012).

In terms of cerebrovascular disease (stroke), a circulating microbial index comprised of 2 bacterial (*Chlamydophila pneumoniae, H. pylori*) and 3 viral (CMV, herpes simplex virus 1 and 2) species was associated with a 40% increased risk of stroke (Elkind et al., 2010). Furthermore, various bacteria, parasites, fungi, and viruses, including *H. pylori, Trypanosoma cruzi, Asperigillus*, CMV, and others have been implicated in stroke pathogenesis (Miller and Elkind, 2016). One way that microbial burden may contribute to stroke risk is by the binding of LPS to fibrinogen. Within 10 min of exposure of fibrinogen to LPS, fibrils and dense, matted deposits are formed (Pretorius et al., 2016). Similarly, the addition of LPS to whole blood results in amyloid-like matted deposits and a hypercoagulated state (i.e., blood clots), evidence that provides a link between circulating microbial burden with stroke.

In addition to LPS's causative role on insulin resistance, elevated circulating LPS is associated with a significantly higher risk for the development of type 2 diabetes in middle-aged adults (Pussinen et al., 2011). LPS may not be the only bacterial trigger for inducing insulin resistance. In support of this, blood levels of bacterial DNA are elevated in middle-aged, and in older adults with type II diabetes, when compared with healthy controls (Amar et al., 2011; Sato et al., 2014).

If we are fortunate to avoid these diseases and live to achieve centenarian status, infectious disease as a major cause of death arises again. In Japan, more than 40% of all centenarian deaths are due infectious diseases, including pneumonia (Tauchi et al., 1999). Similarly, in a larger study of ~36,000 centenarians from the UK, other than “old age,” the leading cause of death was pneumonia (Evans et al., 2014). In short, over the past 115+ years, we haven't eliminated the adverse effects of microbes on our health, we've merely delayed them!

As an argument against the role of microbes on causing many aspects of aging and age-related disease, it is important to note that host aging does indeed occur in their absence. Although lifespan in microbe-free mice is increased by 20–50% (Gordon et al., 1966; Tazume et al., 1991), these animals are not immortal. Nonetheless, as presented here, microbes are involved in mechanisms related to aging and age-related disease, and accordingly, I posit that any classification of aging as a disease should include the contribution of microbes.

## Author contribution

The author confirms being the sole contributor of this work and approved it for publication.

### Conflict of interest statement

The author declares that the research was conducted in the absence of any commercial or financial relationships that could be construed as a potential conflict of interest.
